# Bending Dynamics of Magnetic Filaments at a Curved Bacterial Bath Interface

**DOI:** 10.1021/acs.langmuir.5c04978

**Published:** 2025-12-12

**Authors:** Mehdi Shafiei Aporvari, Sabareesh K. P. Velu, Ali-Reza Moradi, Emine Ulku Saritas

**Affiliations:** † UNAM − National Nanotechnology Research Center, Bilkent University, Ankara 06800, Turkey; ‡ National Magnetic Resonance Research Center (UMRAM), Bilkent University, Ankara 06800, Turkey; § Department of Physics, 708335 Rathinam College of Arts and Science, Coimbatore, Tamil Nadu 641021, India; ∥ Department of Physics, 113403 Institute for Advanced Studies in Basic Sciences (IASBS), Zanjan 45137-66731, Iran; ⊥ School of Quantum Physics and Matter, Institute for Research in Fundamental Sciences (IPM), Tehran 19395-5531, Iran; # Department of Electrical and Electronics Engineering, Bilkent University, Ankara 06800, Turkey

## Abstract

The bending dynamics of microscopic filaments play a crucial role in various mechanical and biological processes. While thermal fluctuations typically have a minor effect, active fluctuations can provide sufficient mechanical energy to alter bending deformations in these systems. In this work, we investigate how active noise influences the bending dynamics of a self-assembled chain of magnetic particles at a curved liquid–air interface in the presence of swimming *E. coli* bacteria. We analyze the bending behavior of semiflexible chains under a lateral gravitational force and compare their response in passive and active baths. In a passive bath, the chain remains bent, fluctuating slightly around its deformed configuration due to thermal noise. However, in an active bath, strong bacterial activity suppresses the average bending deformation, causing the chain to fluctuate around a nearly straight configuration. These findings highlight the impact of active fluctuations on self-assembled microstructures and demonstrate a simple yet effective approach to studying the mechanical dynamics of microscopic filaments.

## Graphical abstract

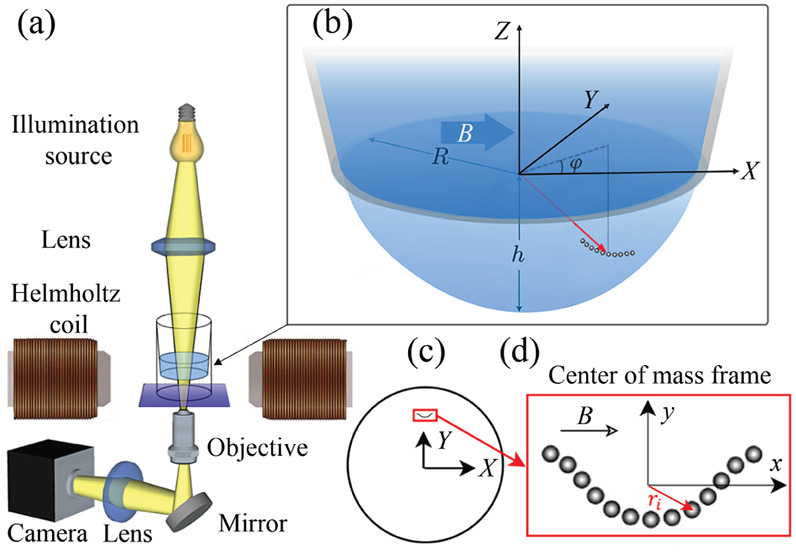

## Introduction

Many microscopic biological and mechanical systems contain filamentous or elongated objects, such as cytoskeletal microtubules,^1^ actin networks,^2,3^ flagella,^4,5^ and DNA fragments.^6–9^ The bending behavior of these objects play a critical role in the overall dynamics of the system. Since microscopic filaments often interact with complex environments that are far from thermodynamic equilibrium, understanding their mechanical behavior remains an ongoing challenge.

One method to addressing these challenges is to use artificial chains, such as self-assembled chains, which offer a means to explore the fundamental nature of complex biological systems. Magnetic chains, in particular, serve as promising artificial filaments since they can be easily manipulated using external magnetic fields. Besides their potential for studying biological materials, magnetic chains have many applications in artificial swimmers,^10–14^ micromixers,^15–17^ and magnetorheological fluids.^18,19^

Active systems that consist of a large number of autonomous agents are commonly found in living materials.^20^ These agents utilize either their internal energy or energy obtained from the environment and convert it into motion.^21,22^ The dynamics of chain-like filaments with internal degrees of freedom in an active environment differ significantly from those in a passive environment. The behavior of polymer chains and filaments in an active bath, as a function of chain flexibility and bath activity, has been theoretically investigated in many studies.^23–28^ It has been demonstrated that the activity of the system can lead to to various intriguing effects, such as nonuniform pressure along curved filaments and ratchet currents^29^ unusual swelling behavior in polymers,^30–32^ nonmonotonic diffusivity,^33^ and facilitation of polymer looping.^34^

An important feature of filamentous objects is their rich mechanical properties, which govern their response to external forces and environmental conditions. In microscopic filaments, the bending dynamics strongly depend on the surrounding environment, particularly for biological filaments that naturally exist in complex environments. For example, microtubules become stiffened when embedded in an elastic medium. allowing them to withstand compressive loads up to 2 orders of magnitude higher than a free microtubule.^1^ Similarly, thermal forces influence shape and orientation fluctuations of linked colloidal chains.^35^

In nanoscale systems, the influence of thermal noise on the bending dynamics becomes more significant. For example, in the case of nanoscale filaments, the critical buckling load increases with increasing temperature due to thermal fluctuations, which assist in the healing mechanism of buckling deformation.^36–38^ In other words, thermal fluctuations enhance the mechanical stability of nanofilaments. In microscopic systems, thermal fluctuations at room temperature usually have only a minor effect on mechanical bending, since the bending rigidity of the system is much larger than the thermal energy *k*_*B*_ *T*.^37–39^ However, in some microscopic systems where an effective temperature can be defined, changes in the effective temperature can lead to strong mechanical bending. For instance, slender self-assembled domains of magnetic colloids formed in an external magnetic field have been shown to bend when the magnetic field changes suddenly.^40^ Similarly, in active systems, the concept of an effective temperature is sometimes used to describe dynamics that deviate significantly from thermodynamic equilibrium.^41^ This high effective temperature suggests that the overall fluctuations in active systems can be strong enough to significantly modify the mechanical properties of micron-sized filaments.

In this work, we experimentally demonstrate that an active bath effectively suppresses the average bending deformation of microscopic semiflexible chains. The experiments are conducted at the liquid–air interface within a hanging droplet, where microscopic chains are formed by self-assembling superparamagnetic colloids using an adjustable external magnetic field. We first perform experiments in a passive bath to investigate the effect of thermal fluctuations and lateral gravitational forces on the bending dynamics of self-assembled chains at a curved interface. The results are then compared with experiments conducted in an active bath containing cultured *E. coli* bacteria.

## Experimental Details

Figure 1 shows a schematic of the experimental setup used to manipulate the superparamagnetic chains at the curved liquid–air interface of a hanging droplet. The system was imaged by a homemade inverted microscope with a 20X, 0.5 NA objective (Nikon, N20X-PF). The images were then recorded using a CMOS camera (Thorlabs, DCC1645C).

**Figure 1:**
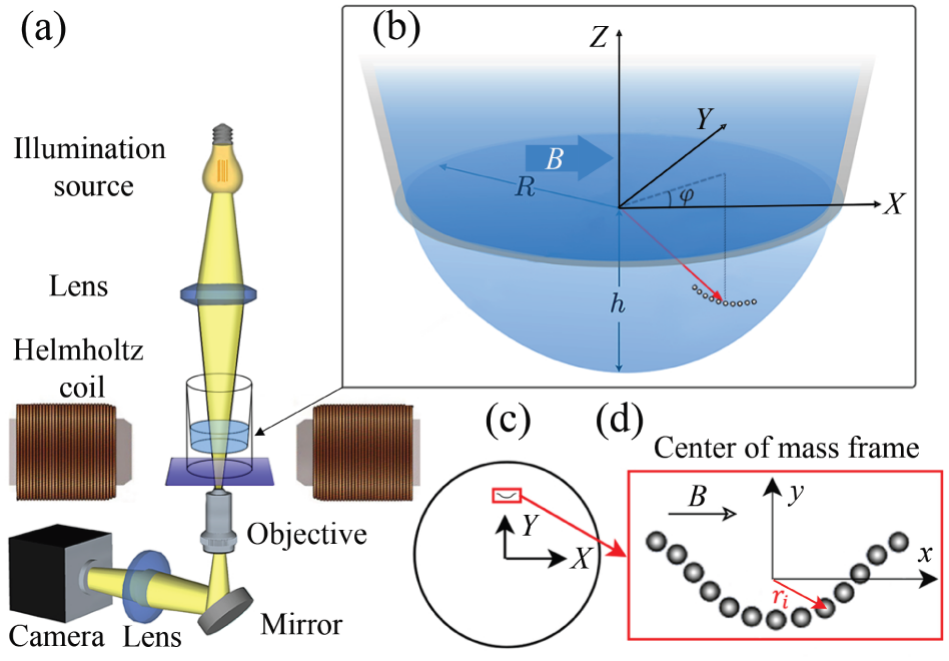
(a) Schematic of the experimental setup, which contains an electromagnet and an inverted microscope. For illustrative purposes, the sample cell is not to scale. (b) A magnified view of the hanging droplet showing an isolated chain sedimenting on the liquid–air interface under a magnetic field applied in the *x* direction. The chain is positioned at an azimuthal angle ϕ. Gravity exerts a tangential component along the interface, causing the chain to bend toward the bottom of the droplet. (c) Camera view of the droplet from below. In this work, the chain is initially positioned slightly away from the center of the droplet, mainly at an angle ϕ = 90°. (d) Enlarged view of the chain in center of mass frame.

In order to investigate the chain dynamics at curved interfaces, we performed our experiments on the liquid–air interface formed at the hanging droplet using our previously described sample cell design.^41^ As schematically shown in Figure 1a, magnetic colloidal particles (microparticles PS-MAG-S2180, diameter *d*_0_ = 3.9 μm, density 1.62 g/cm^3^, mass susceptibility 2.5 × 10^–4^ m^3^/kg) sediment on the lower liquid–air interface of the droplet due to gravity. The details of droplet formation in the capillary tube are provided in the Supporting Information, where it is shown that the hanging droplet in our experiment approximately forms a spherical curvature. The curvature of the interface can be adjusted by varying the volume of the liquid in the droplet.

To form isolated self-assembled chains, a dilute solution of magnetic particles was used in the hanging droplet. Once the particles sedimented at the bottom liquid–air interface, an external magnetic field was applied. Chains formed due to dipole–dipole magnetic interactions between neighboring particles.^41^ In our experiments, even at the smallest applied magnetic field (*B* = 0.7 mT), the contact energy between superparamagnetic beads is approximately 26 *k*_*B*_ *T*, which is well above the thermal energy and sufficient to support chain formation (see Supporting Information for details). Here, we define chain length, *N*, as the number of particles in the chain. The chain length varied between *N* = 2 and *N* = 20. The flexibility of the chains was controlled by adjusting the magnetic field strength. Also, this frictionless liquid–air interface allows the magnetic beads to assemble under a significantly lower magnetic field compared to a liquid-glass interface. In this work, an isolated chain is selected to study the bending dynamics under gravitational force. A magnified view of the droplet within the sample cell is shown in Figure 1b, where the magnetic field is aligned along the *X* direction and the gravitational force acts along the *Z*-direction. During experiments, chains remain sedimented at the liquid–air interface Figure 1b shows a chain at the droplet interface, located at an azimuth angle of ϕ and at a distance *d* from the *Z*-axis. Due to gravity, the chain moves toward the central point at the bottom interface of the droplet (in other words, the chain moves toward the *Z*-axis while remaining at the liquid–air interface). Unless otherwise mentioned, in our experiments, the interface radius of curvature is much larger than the *R*, and the chain is initially positioned at an azimuthal angle ϕ = 90° (Figure 1c). Experimentally, the position of each particle in the chain is determined using digital video microscopy.

The rigidity of the self-assembled chains is controlled by an external magnetic field. The chains in our experiments are in semi-flexible regime. Note that, unlike permanent filaments^39^ the magnetic field in self-assembled chains cannot be reduced below a certain level to achieve more deflected configurations due to the lack of connections between the particles. There is a critical magnetic field strength below which the self-assembled chains will disassemble. The precise value of this critical field strength depends on various factors, such as the particle size and structure, the surrounding fluid, and even the length of the chain. Additionally, the critical strength is influenced by whether the chain is suspended in an active bath or a passive bath. Our experiments were performed above the critical field strength to prevent the chain from disassembling. The experiments were conducted in both passive and active baths. The critical field strength was determined by gradually reducing the magnetic field until the chains began to disassemble. The lowest magnetic field at which a chain of 17 particles remained connected was approximately 2.1 mT in the active bath and 0.6 mT in the passive bath. For the passive bath, we used double-distilled water, and for the active bath, we employed motile *E. coli* (wild-type strain RP437) bacteria cultured using a standard double-step protocol in a motility buffer.^41,42^

It is important to note that the colloidal particles in our experiments do not adsorb onto liquid–air interface, where a three-phase contact between the liquid, air, and solid particle would form. When a colloidal particle becomes adsorbed at the interface, the energy required to remove it (i.e., fully immersing it into the liquid) can be significant, making the adsorption effectively irreversible. However, based on our observation, the particles in our experiments do not exhibit such adsorption. This is evident from the fact that we can readily move the particles away from the interface by increasing the upward magnetic field, applied in the *z*-direction using a permanent magnet placed above the sample with an adjustable distance *d*. In our case, the particles are initially suspended in the liquid and remain in the liquid when they sediment. The equilibrium position of the particles near the interface is determined by the balance between gravity and the upward electrostatic forces from the interface. Furthermore, due to the high surface tension of water, any deformation of the liquid–air interface caused by the presence of sedimented microparticles is negligible.^43^

## Results and Discussion

To study the bending dynamics of a microfilament, we analyze a magnetic chain that has sedimented on a curved liquid–air interface. Specifically, we consider a location where the chain is positioned at an azimuthal angle ϕ = 90° (see Figure 1c). At this orientation, the tangential component of the gravitational force along the interface is perpendicular to the applied magnetic field. This causes the chain to drift toward the center of the droplet while remaining at the interface. As a result, the chain drifts toward the center of the droplet while remaining confined to the interface. Because the central particles experience different hydrodynamic disturbances than the particles at the ends, the chain bends. This behavior resembles the sedimentation of flexible chains in bulk liquid.^43^ To create a curved interface, we used droplets with a radius of curvature of *r*_*c*_ = 11.3 mm in these experiments, providing a slight slope that allows the chain to gradually move toward the bottom center of the droplet (see Supporting Figure S1 for more details). For simplicity, we study the bending dynamics of the chain in the two-dimensional center of mass (COM) frame, denoted as the *xy*-plane (Figure 1d). The position of a particle in the chain with respect to the COM frame can be written as **r**_*i*_(*t*) = (*x*_*i*_(*t*),*y*_*i*_(*t*)). Figure 2 shows the bending of a magnetic chain under different magnetic field strengths in motility buffer (*E. coli* buffer). As seen in the figure, at high magnetic fields the bending is negligible. However, at very low magnetic fields, the chain bends completely in the direction of the tangential gravitational force.

**Figure 2:**
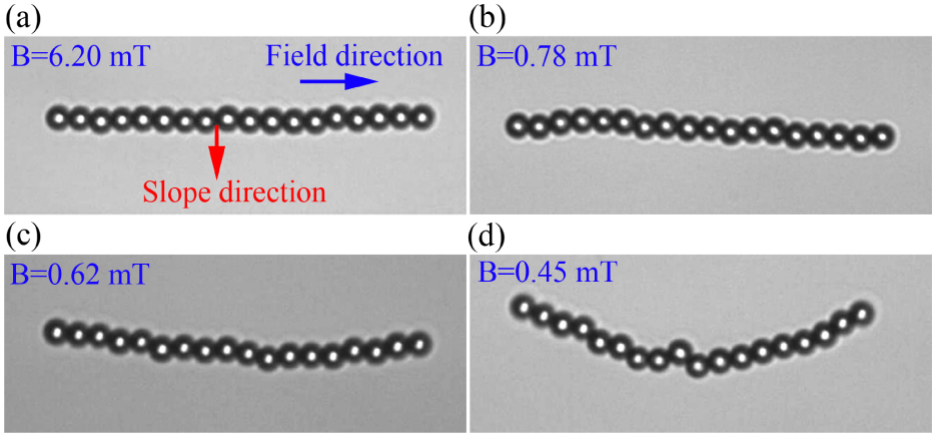
Bending of magnetic self-assembled chains at a curved liquid–air interface under lateral gravitational force, shown for different external magnetic field strengths. (a) *B* = 6.20 mT, (b) *B* = 0.78 mT, (c) *B* = 0.62 mT, and (d) *B* = 0.45 mT. Under weak magnetic fields, the chain bends more in the direction of the lateral gravitational force. The liquid is a motility buffer (bacterial environment). The droplet curvature is *r*_c_ = 11.4 mm. Blue and red arrows indicate the directions of the external magnetic field and the tangential gravitational force, respectively.

The presence of salt in the motility buffer reduces electrostatic repulsion between beads, increasing the likelihood of permanent bead–bead adhesion when particles are brought into contact under strong magnetic forces. For simplicity, and because particle sticking in motility buffer makes it difficult to analyze small chain deflections, in the following we conducted passive-case experiments in pure water. To record chain fluctuations long enough for analysis, we conducted the experiments in water at a larger interface radius of curvature, i.e., *r*_*c*_ = 17.2 mm. This allowed the chain to remain in the field of view longer before drifting out due to gravity. Figure 3 shows the deflection curves *y*_*i*_(*t*) for a chain of 16 superparamagnetic particles at the water–air interface in a passive bath, plotted at equal time intervals (blue curves) in the COM frame under different magnetic fields (see Supporting Movie S1). Each particle in the chain undergoes positional fluctuations due to thermal noise, and the chain as a whole experiences small-angle rotations around its center of mass. These effects naturally broaden the instantaneous distribution of particle positions, especially for particles at the chain ends, which are less constrained than those near the center. Although the stochastic motions of each magnetic particle in the chain leads to random deflection curves, the average positions of the particles (represented by red dots) form a bent curve. At high magnetic fields, the bending of the chain is small. However, upon close inspection, this subtle bending can still be detected, as illustrated in Supporting Figure S3. As the magnetic field strength decreases, the chain exhibits both larger fluctuates and an increased bending effect.

**Figure 3:**
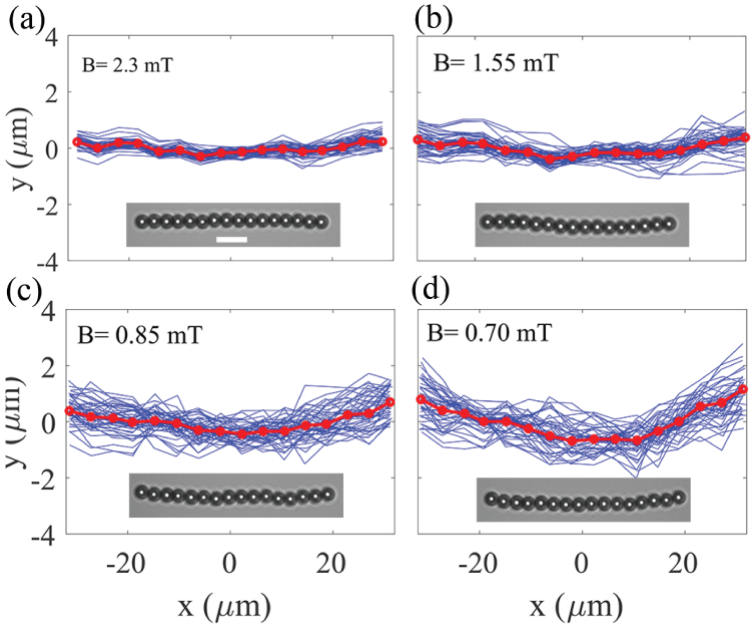
Deflection of a chain with *N* = 16 particles at a curved liquid–air interface in a passive bath under various external magnetic fields. (a) *B* = 2.3 mT, (b) *B* = 1.55 mT, (c) *B* = 0.85 mT, and (d) *B* = 0.70 mT. The curvature of the droplet is *r*_*c*_ = 17.2 mm. The tangential gravitational force at the interface is perpendicular to the magnetic field (and thus to the chain direction), meaning ϕ = 90°. Red dots show the average position of each monomer in the center of mass frame. Under weak magnetic fields, the chain exhibits greater deflections. Insets show example snapshots of the chain for each case. Deflection curves (blue) are plotted at 3 s intervals over a total duration of 120 s. The scale bar is 10 μm.

For comparison, we repeated the experiments in the presence of an active bath as shown in Figure 4 Compared to the passive case, under the same curvature in the active bath, the chains remained within the field of view for longer durations due to strongly anisotropic diffusion—faster along their longitudinal axis and slower in the perpendicular direction.^41^ This allows us to perform experiments at higher gravitational forces in the active bath compared to the passive case. We therefore performed the experiments at a smaller radius of curvature, *r*_*c*_ = 11.4 mm. Note that a smaller radius of curvature—i.e., a steeper interface—corresponds to a larger tangential gravitational force. Consequently, in the absence of activity, the chains would be expected to bend even more compared to the larger radius of curvature used in the passive case (Figure 3).

**Figure 4:**
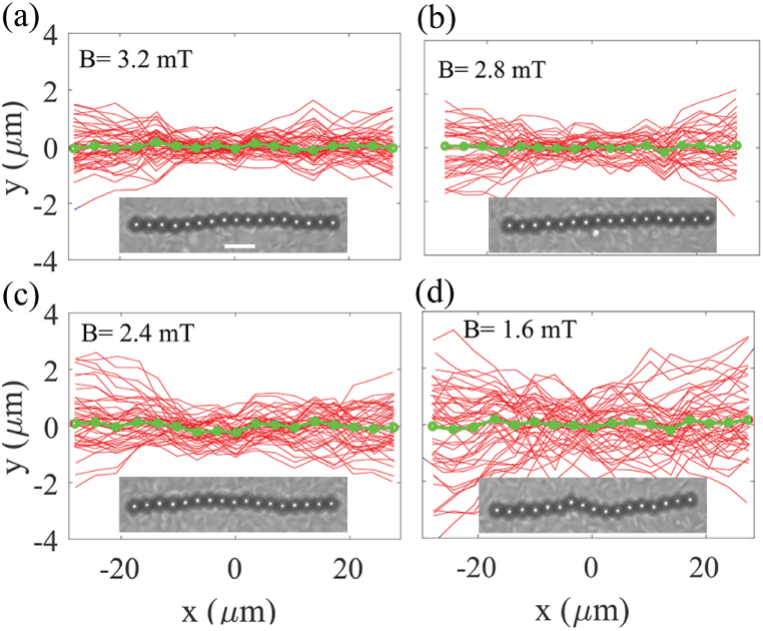
Deflection of a chain with *N* = 17 particles at a curved liquid–air interface in an active bath under various external magnetic fields. (a) *B* = 3.2 mT, (b) *B* = 2.8 mT, (c) *B* = 2.4 mT, and (d) *B* = 1.6 mT. The curvature of the droplet is *r*_*c*_ = 11.4 mm. The tangential gravitational force at the interface is perpendicular to the magnetic field (and thus to the chain direction), meaning ϕ = 90°. The average position of the particles in the chain (green dots) is not deflected, indicating that bacterial activity suppresses the average bending deformation in the active bath. Insets show example snapshots of the chain for each case. Deflection curves (red) are plotted at 1.7 s intervals over a total duration of 80 s. The scale bar is 10 μm.

It is evident that the chain exhibits larger fluctuates compared to the chains in the passive bath, which can be attributed to the activity of the swimming bacteria in the active bath. Surprisingly, the deflected curves are symmetrically distributed, such that the average positions of the magnetic particles (indicated by green dots) form almost a straight line with negligible deformation, demonstrating the suppression of the bending effect (see Supporting Movie S2). It is evident that any deflection within the chain is quickly restored, confirming the bacterial activity suppresses the average bending of the magnetic chain caused by the lateral gravitational force. Similar effects have been reported for nanoscale elastic ribbons in a thermal bath,^36–38^ where thermal fluctuations increase the critical buckling load compared to the classical Euler’s buckling theory. In our system, the influence of thermal noise is minimal or negligible, and it is unable to suppress the buckling deformation in microlength-scaled chains (Figure 3). However, the active noise is strong enough on microlength scaled chains to suppress the buckling effect. The suppression of average bending can be understood in terms of an effective temperature, which is sometimes used to describe active systems. This effective temperature is significantly higher than the thermodynamic temperature in active baths^41^ to the extent that it is capable of overcoming the bending of microchains caused by gravity. We note that in this work we consider the bending dynamics at high bacterial concentration. It is important to recognize that the bacterial bath is inherently a nonequilibrium system, and changing the concentration effectively creates a different state with potentially distinct behavior. For example, at very low concentrations, the active forces may be insufficient to overcome the gravitational bending force. While exploring the dependence of chain bending on bacterial concentration would be an interesting direction for future work, it lies beyond the scope of the present study.

To better understand the bending behavior in our system, we examine the internal dynamics of self-assembled magnetic chains. For simplicity, we focus on the dynamics of these chains at nearly flat liquid–air interfaces. This flat interface is achieved by using smaller liquid volumes in our sample cell, specifically 42 μL in this case, which results in a droplet curvature of *r*_*c*_ = 35.7 mm. In Figure 5, we analyze the root-mean-square (RMS) displacement σ_*i*_(τ), perpendicular to the chain direction for the *i*th particle, defined by^44^

1 $$\sigma_{i}\left(\tau \right)=\langle {[y_{i}(t)-y_{i}(t+\tau )]}^{2}\rangle^{1/2}$$

where *y_i_* (*t*) denotes the deflection of the *i* ^th^ particle along the chain in the direction perpendicular to **B** at time *t* (see Figure 1c), and angular brackets represent an average over all times *t*. This local, time-domain approach is particularly suitable for our short, semiflexible chains in a dynamic, nonequilibrium environment, where standard eigenmode decomposition is less robust. σ­(τ) has previously been shown to exhibit power-law behavior σ­(τ)∝ τ^∝^ for short times, before reaching a plateau value at longer times due to the restriction of the internal motion of each particle in the dipolar potential exerted by the other particles in the chain.^41,44,45^ For a freely diffusing particle, the growth exponent α equals 0.5, whereas for a monomer in a dipolar chain in a passive bath, α is less than 0.5, indicating hindered diffusion due to internal constraints and dipolar couplings between monomers.^41,44^ In an active bath, despite the hindered diffusion of each particle in the chain, the exponent reaches superdiffusive values (α > 0.5) at sufficiently low magnetic fields.^41^ Figure 5(a) shows the root-mean-square (RMS) displacement, σ_*i*_(τ), of the first particle in an 8-particle chain in active and passive baths under a magnetic field of *B* = 3.9 mT. In the passive bath, α < 0.5, indicating subdiffusive behavior, whereas in the active bath, α > 0.5, consistent with superdiffusive motion. Lag times <0.4 s is used to obtain α by fitting.

**Figure 5:**
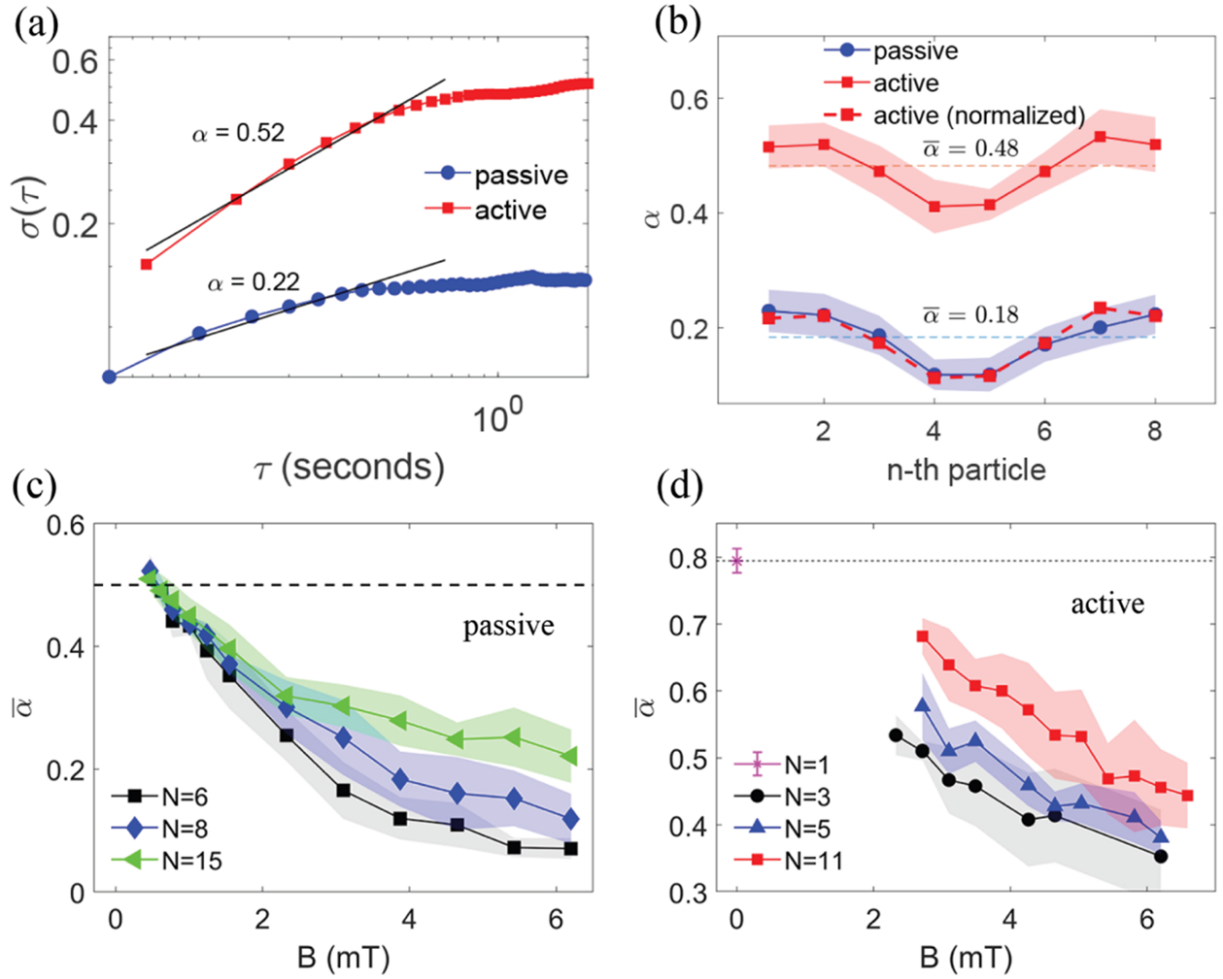
Internal chain dynamics in active and passive baths. (a) Root-mean-square (RMS) displacement, σ_*i*_(τ), of the first particle in an 8-particle chain in active and passive baths under a magnetic field of *B* = 3.9 mT. The curvature of the droplet is *r*_*c*_ = 35.7 mm, creating an approximately flat interface. (b) Growth exponent, α, for each particle of the 8-particle chain in a passive bath (blue circles) and an active bath (red squares, solid lines) under the same field. Dashed lines indicate the average growth exponent, α̅. The red squares with dashed lines show normalized α values for the active bath, obtained by applying a constant offset equal to the difference between the average growth exponents of the active and passive cases. (c, d) α̅ as a function of chain length and magnetic field strength for (c) the passive bath (water) and (d) the active bath. Horizontal dashed lines in (c) and (d) mark the values for a free particle in water (passive bath) and in bacterial suspension (active bath), respectively.

Figure 5b shows the growth exponent, α, for each particle of the 8-particle chain in a passive bath (blue circles) and an active bath (red squares) under the same field. As seen, α reaches its largest values for particles near both ends of the chain and is significantly reduced for the central monomers in both active and passive baths. In other words, central particles are more hindered, while the particles at both ends fluctuate more freely.

The trends for both baths are qualitatively similar; however, the growth exponent in the active bath is more than twice as large as in the passive bath. Normalizing the active bath α values by applying a constant offset equal to the difference between the average growth exponents of the active and passive cases shows that the active and passive data overlap closely (red squares with dashed lines in Figure 5b). This suggests that, in the active case, α can be expressed as the sum of two contributions: one that depends on the internal position of particles in the chain but is independent of activity, and another that depends on activity but is independent of internal particle positions.

In Figure 5c,d, α̅, the average of α among all particles in a chain, is shown as a function of magnetic field strength. For passive bath (Figure 5(c)), we see the expected behavior of α̅ < 0.5, which approaches 0.5 at low magnetic fields. We observe that α increases with chain length, indicating that monomers in longer chains exhibit greater transverse fluctuations. This behavior is consistent with the well-known tendency of longer elastic filaments to be more susceptible to bending.^46^ This effect is examined in more detail in the Supporting Information by considering thermal expansion in self-assembled chains of varying lengths. We see the same behavior for chains in active bath in Figure 5d; however, here α̅ is much larger than its corresponding values in the passive bath, even exceeding the free diffusion value of 0.5. Note that, in the active case, we cannot decrease the magnetic field to very small values because self-assembled chains tear apart at low fields due to the high activity. There are two potential ways to address this gap at low magnetic fields: One is by reducing the activity of the bath through dilution of the bacteria concentration, which brings the system closer to the regime of a passive bath at low fields, as shown in Figure 5c. The other approach is by using permanent chains, where beads are attached to each other using ligands. In the latter case, within the flexible polymer regime, where monomers can freely rotate against each other, computational studies have shown that activity tends to cause flexible polymer chains to swell.^30–34^ Even though our chains are not flexible but semiflexible, we observed similar behavior, where the magnetic chains, on average, tend to form a straight line in active bath.

Next, we examine the crowding effects on the bending of the chains on a curved interface in Figure 6. Since dead or inactive *E. coli* in the motility buffer tend to aggregate and adhere to the colloidal chains, we used small nonmagnetic particles (silica, 0.922 μm) as crowding agents alongside the magnetic particles in the sample. The size of nonmagnetic particles is comparable to that of average *E. coli* bacteria. The magnetic field for all cases is *B* = 0.8 mT, and the droplet curvature is 11.4 mm. As shown in Figure 6a,b, when the direction of the chain aligns with the slope at the liquid–air interface (ϕ = 180°), the chain stays straight. However, when the directions of the chain and the slope are perpendicular (ϕ = 90°), the chain bends due to the gravitational force in both uniform and crowded environments (Figure 6c,d). In other words, unlike the case of an active crowded bath, a passive crowded environment does not change the bending configurations in our system.

**Figure 6:**
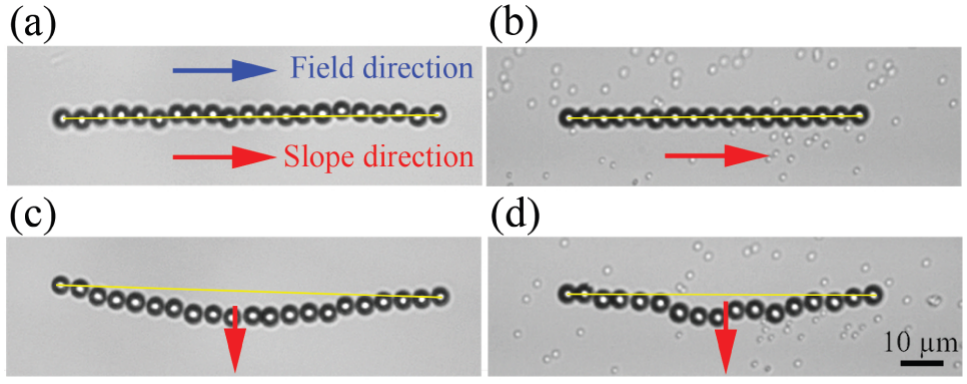
Influence of crowdedness on chain deformation at curved liquid–air interface. The curvature of the droplet is *r*_*c*_ = 11.4 mm and *B* = 0.8 mT. The chain configuration when the magnetic field and the component of gravitational force on tangent plane at the interface (slope direction) are in the same direction (ϕ = 180°) is shown (a) in pure water and (b) in a crowded environment with 1 μm sized colloids. The chain configuration when the magnetic field and slope are perpendicular (ϕ = 90°) is shown (c) in pure water and (d) in a crowded environment. The red arrows indicate the direction of the slope at the curved liquid–air interface, and the blue arrow shows the magnetic field direction. The chain remains straight when oriented along the slope direction. In contrast, if the chain is oriented perpendicular to the slope direction, it buckles due to the lateral gravitational forces, whether the environment is pure water or crowded with colloids.

Finally, it is worth noting that another mechanism that can lead to bending deformation of a self-assembled chain is rapid thermal expansion. Although this is a completely different mechanism, unrelated to the bending we observe due to gravitational drag, it provides a simple system to study how the length of a self-assembled chain affects its bending. In this case, an expanding force is applied along the chain due to the thermal expansion, which creates excess pressure along the chain and causes it to bend laterally. Thermal expansion could be introduced into our system through rapid changes in the magnetic field. This effect is discussed in the Supporting Information, where a rapid decrease in the magnetic field results in bending deformation of the chains on a flat liquid–air interface.

## Conclusions

In summary, we experimentally studied the bending dynamics of a microchain in both active and passive baths. We demonstrated that the bending of a self-assembled chain due to a lateral force can be suppressed in active bath. While in passive bath at a curved interface, a self-assembled chain fluctuates slightly around its bent configuration due to the thermal noise, in an active bath, strong active noise suppresses the bending, causing the chain to fluctuate around a straight configuration. This work also presents a simple technique for studying the mechanical dynamics of microfilaments using self-assembled magnetic chains, which is of great importance for many biological intracellular interactions.

## Supplementary Material







## Notes

Supporting Information: magnetic dipole–dipole interaction; droplet formation at the end of a narrow vertical tube. Bending deformation of self-assembled chains due to thermal fluctuations (PDF)
Movie S1: Time series of a self-assembled magnetic chain in a passive bath (AVI)
Movie S2: Time series of a self-assembled magnetic chain in an active bath (AVI)

## ∇

Department of Physics, University of Illinois, Chicago, IL 60607, USA
