# Supplementary material for: Bending Dynamics of Magnetic Filaments at a Curved Bacterial Bath Interface
Source: Langmuir. 2025 Dec 12;41(50):34054–60. doi: 10.1021/acs.langmuir.5c04978 (PMC12751016; doi:10.1021/acs.langmuir.5c04978)
Supplement: Supplementary file 1 [file la5c04978_si_001.pdf]

Supporting Information

Bending dynamics of magnetic filaments at a curved bacterial bath interface

Mehdi Shafiei Aporvari,[*,†,‡,¶] Sabareesh K. P. Velu,[§] Ali-Reza Moradi,[‖,⊥] and Emine Ulku Saritas[*,#,‡,¶]

†*Present address: Department of Physics, University of Illinois, Chicago, IL 60607, USA.*

‡*UNAM – National Nanotechnology Research Center, Bilkent University, Ankara 06800, Turkey*

¶*National Magnetic Resonance Research Center (UMRAM), Bilkent University, Ankara 06800, Turkey*

§*Department of Physics, Rathinam College of Arts and Science, Coimbatore 641021, Tamil Nadu, India*

‖*Department of Physics, Institute for Advanced Studies in Basic Sciences (IASBS), Zanjan 45137-66731, Iran*

⊥*School of Quantum Physics and Matter, Institute for Research in Fundamental Sciences (IPM), Tehran 19395-5531, Iran*

#*Department of Electrical and Electronics Engineering, Bilkent University, Ankara, Turkey*

E-mail: mshphy@gmail.com; saritas@ee.bilkent.edu.tr

Table of Contents

1. Magnetic dipole-dipole interaction S2
2. Droplet formation at the end of a narrow vertical tube S3

3. Bending deformation of self-assembled chains due to thermal fluctuations S4
4. Figure S1 . . . . S6
5. Figure S2 . . . . S7
6. Figure S3 . . . . S8
7. Movie S1 . . . . S8
8. Movie S2 . . . . S8
9. References S9

Magnetic dipole-dipole interaction

The dipole–dipole interaction energy between two magnetic dipoles $\mathbf{m}_1$ and $\mathbf{m}_2$ separated by victor $\mathbf{r}$ is:

$$U = \frac{\mu_0}{4\pi r^3}[\mathbf{m_1} \cdot \mathbf{m_2} - 3(\mathbf{m_1} \cdot \hat{r})(\mathbf{m_2} \cdot \hat{r})] \tag{S1}$$

For identical particles with magnetic moments aligned by an external field, and assuming contact at a center-to-center distance $r = d$, the contact energy simplifies. For the case where dipoles aligned along the axis between particles (head-to-tail alignment):

$$U_{contact} = -\frac{\mu_0 m^2}{2\pi D^3} \tag{S2}$$

For the case where dipoles perpendicular to the line joining centers (side-by-side alignment):

$$U_{contact} = \frac{\mu_0 m^2}{2\pi D^3} \tag{S3}$$

So, the attractive contact energy (minimum energy) is:

$$U_{min} = -\frac{\mu_0 m^2}{2\pi D^3} \tag{S4}$$

In our case, the dipole moment $\mathbf{m}$ is induced by an external magnetic field $\mathbf{B}$, then:

$$m = \chi V B/\mu_0 \tag{S5}$$

where, χ is the magnetic susceptibility, $V = \frac{4}{3}\pi(\frac{d}{2})^3$ is the volume of the particle, and B is the magnetic field strength. Therefore, the contact energy in k_BT is:

$$\frac{U_{contact}}{k_BT} = -\frac{\chi^2 V^2 B^2}{2\pi\mu_0 d^3 k_BT} \tag{S6}$$

where k_B is the Boltzmann constant. For superparamagnetic beads a few k_BT is sufficient to form chains. For the smallest magnetic field used in our experiments, i.e., $B = 0.7$ mT, the contact energy approximately is 26 k_BT.

Droplet formation at the end of a narrow vertical tube

A droplet with a volume size between $42 - 90\mu$L was formed at the end of a small circular tube with an inner radius of $R \approx 2$ mm Fig S1 (a). The gravitational force causes the droplet to hang with nearly spherical shape[1]. To have different interfaces with different slopes, the hanging height, h, was changed by adjusting the volume of the droplet. An image of the lateral view of a droplet of 70 μL water is shown in Fig. S1 (b). As can be seen in Fig. S1 (c), the height of the droplet shows a relatively linear behavior as a function of its volume. While it was not possible to directly measure the height of droplets at very small volumes, the linear fit of the data shows that the height approaches zero at a volume of approximately 39 μL, below which the surface curvature changes sign. The sign change can also be confirmed by considering the motion of sedimented particles at the droplet surface with different water volumes. In fact, for droplet volumes less than approximately 39 μL, the colloidal particles did not aggregate at the center of the interface, instead they moved towards the tube wall.

Bending deformation of self-assembled chains due to thermal fluctuations

Here, we consider a passive bath and examine bending deformation of self-assembled chains due to thermal fluctuations in nearly flat liquid-air interfaces. Specifically, we analyze the chain dynamics at the central region of the bottom interface of a droplet with a radius of curvature $r_c = 35.7$ mm. In this region, the interface is approximately flat, and any drift in particle motion due to gravity is considered negligible (therefore, the angle ϕ does not have any significance in this experiment). Supplementary fig. S3 (a) shows buckling of self-assembled magnetic chains with different lengths ($N = 11$, $N = 19$, and $N > 25$) in water by decreasing the magnetic field from 6.2 mT to zero in 45 seconds. Decreasing the field causes an expansion of the chain and once the internal stress between the particles in the chain increases to values higher than the critical buckling load, the chain deflects in the lateral direction. When the strength of the magnetic field is high, the particles will not fluctuate substantially in the lateral direction, which leads to a straight configuration for the chain. In low magnetic fields, on the contrary, the particles in the chain have more freedom to move laterally. Comparing the expansion of chains with different lengths, we observed that shorter chains ($N = 11$) exhibit slight buckling due to longitudinal expansion. In contrast, longer chains ($N = 19$) show more pronounced buckling compared to the shorter chains. However, when considering very long chains ($N > 25$), as shown in the third column of Fig.S3 (a), the chain initially resists buckling as the magnetic field strength is reduced, but it tears apart at very low magnetic fields due to the high expansive forces acting along the chain as a result of its large thermal expansion.

Supplementary Fig. S3 (b) illustrates the relationship between the magnetic field and the change in the mean distance between the neighbouring particles normalized to the particle diameter d_0. Here, the mean distance is defined as $\Delta d = \langle|\mathbf{r}_{i+1}(t) - \mathbf{r}_i(t)|\rangle$, where $\langle\cdots\rangle$ indicates average over time and particle index. It should be noted that, for each magnetic field

strength, the mean distance is calculated at equilibrium. This means that the calculations are performed after a significant amount of time has passed since the application of the magnetic field, allowing the chain to reach a steady state. Note that this condition is different than those in Supplementary Fig. S3 (a). As can be seen, the increase in the normalized mean center-to-center particle separation, $\Delta d/d_0$, shows a nonlinear behavior with respect to the field magnitude. However, Δd is independent of the chain length within the experimental error. Note that the center-to-center particle separation does not depend on the chain length, but the total chain expansion, which is equal to $(N-1)\Delta d$, does. Recalling the expansion of railroad tracks, the expansion per unit length is the same for both long and short tracks. However, longer tracks are more prone to buckling due to their larger total expansion.

The effect of the magnetic field may be considered by introducing an effective temperature for the thermal bath, where the temperature is expressed as a function of the magnetic field strength[2] (note that the effective temperature defined here differs from the one discussed in the main text. In the main text, the effective temperature is defined based on the degree of activity of the system in a non-equilibrium active bath.). This effect is similar to the railroad buckling caused by thermal expansion. The required internal stress between the particles of self-assembled chains is provided by a huge thermal expansion (exceeding 10% at low magnetic fields, as shown in Fig. S3), which in turn, leads to the buckling instability.

The greatness of this expansion may be understood by the following example: for a 10% length increase of a solid bar with linear expansion of steel in room temperature ($\alpha = 13 \times 10^{-6}$), supposing that the expansion coefficient is independent of the temperature, we would need to increase the temperature to about 7700 °C[3] (i.e. higher than 5500 °C, the temperature at the sun surface).

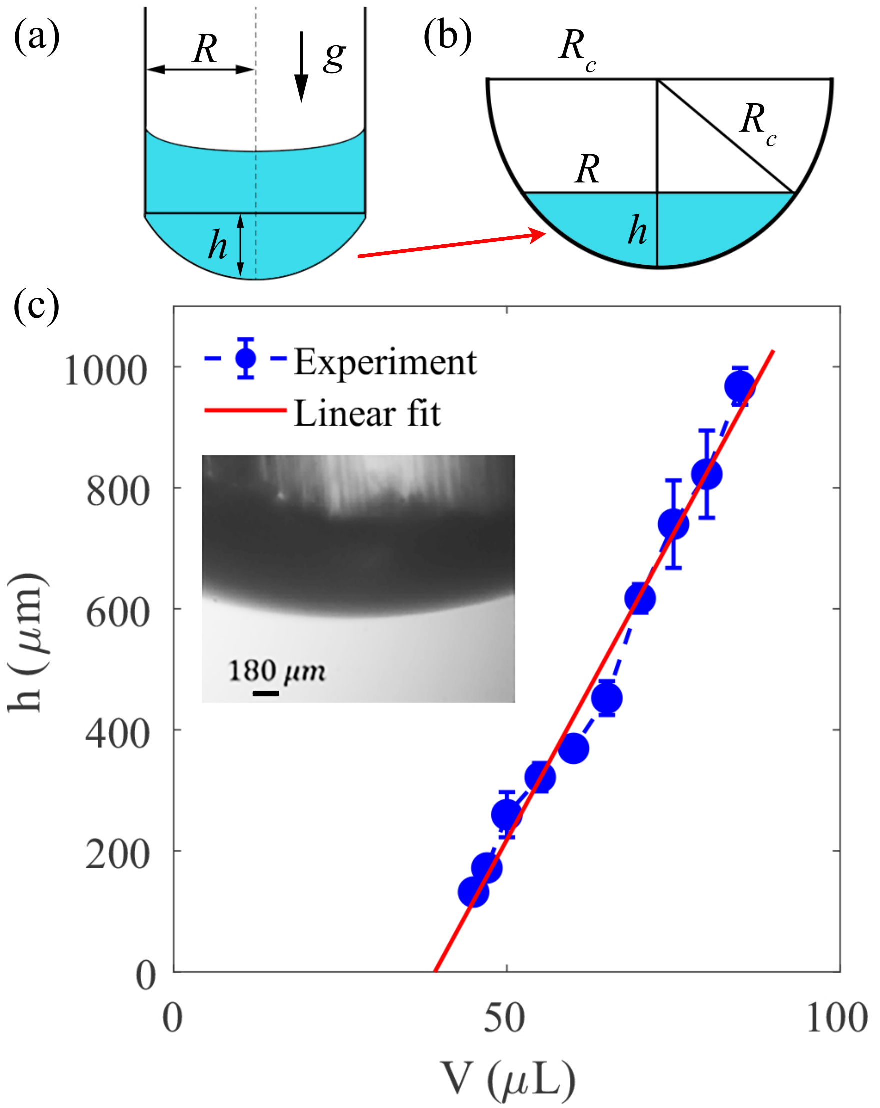

Figure S1: Droplet formation at a vertical capillary tube. (a) Schematic of a droplet hanging from the end of a vertical capillary tube. The droplet height is denoted by h, and the inner radius of the tube is $R \approx 2$ mm. (b) The hanging droplet approximately forms a spherical interface with radius $R_c = (R^2+h^2)/2h$. (c) Droplet height as a function of volume, measured from lateral view images and compared to an image of an empty tube. Blue dots represent experimental data, and the red line is a linear fit given by $h = 20.2V - 792.3$. Note that the hanging height becomes zero at $V \approx 39.2$ μL; for smaller volumes, the curvature of the bottom interface reverses. The inset shows a lateral view image of a 70 μL water droplet.

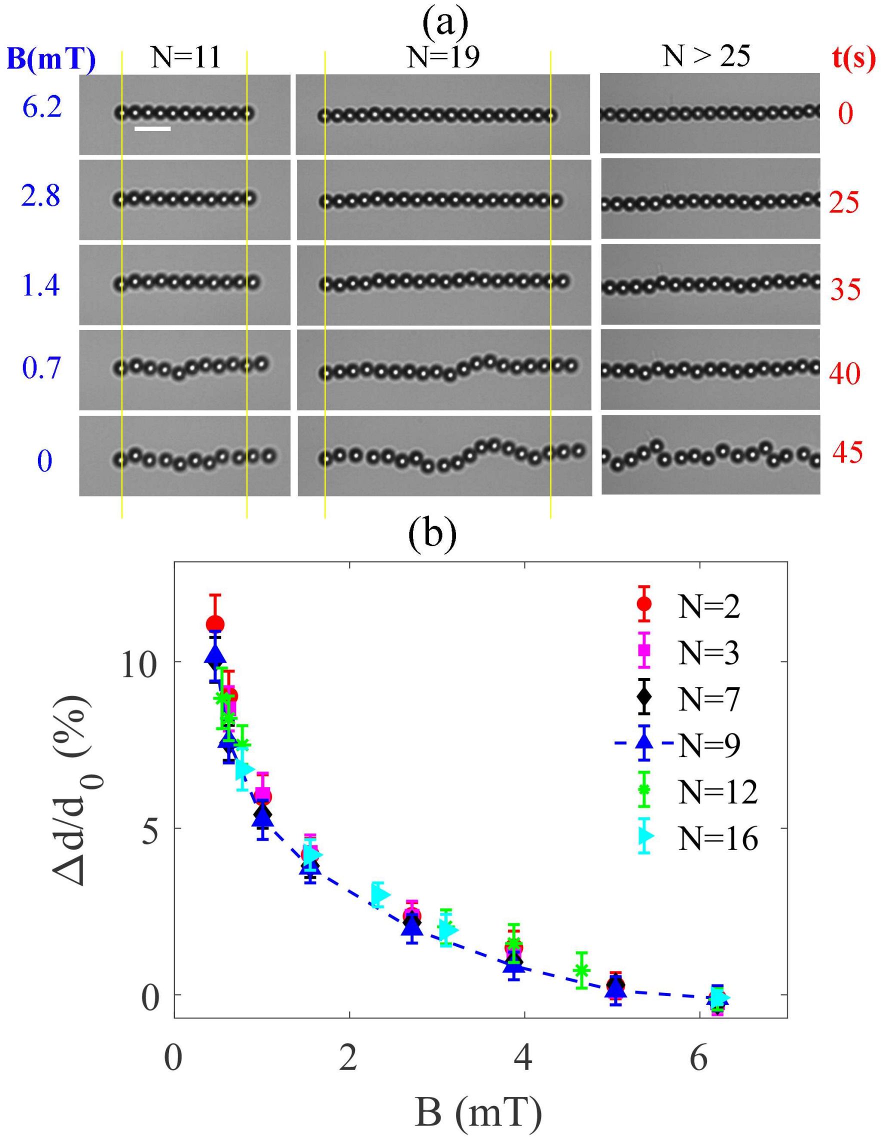

Figure S2: Buckling of self-assembled chains in passive bath due to thermal expansion. (a) Snapshots of self-assembled magnetic chains of different lengths (N) at a nearly flat water-air interface while the external magnetic field is decreased from 6.2 mT to zero in 45 seconds. The volume of the droplet is 42 μL. The scale bar is 10 μm. (b) Relative increase in the mean distance between adjacent particles in chains of different lengths as a function of the magnetic field, with respect to the touching distance d_0, which is equal to the diameter of the magnetic particles.

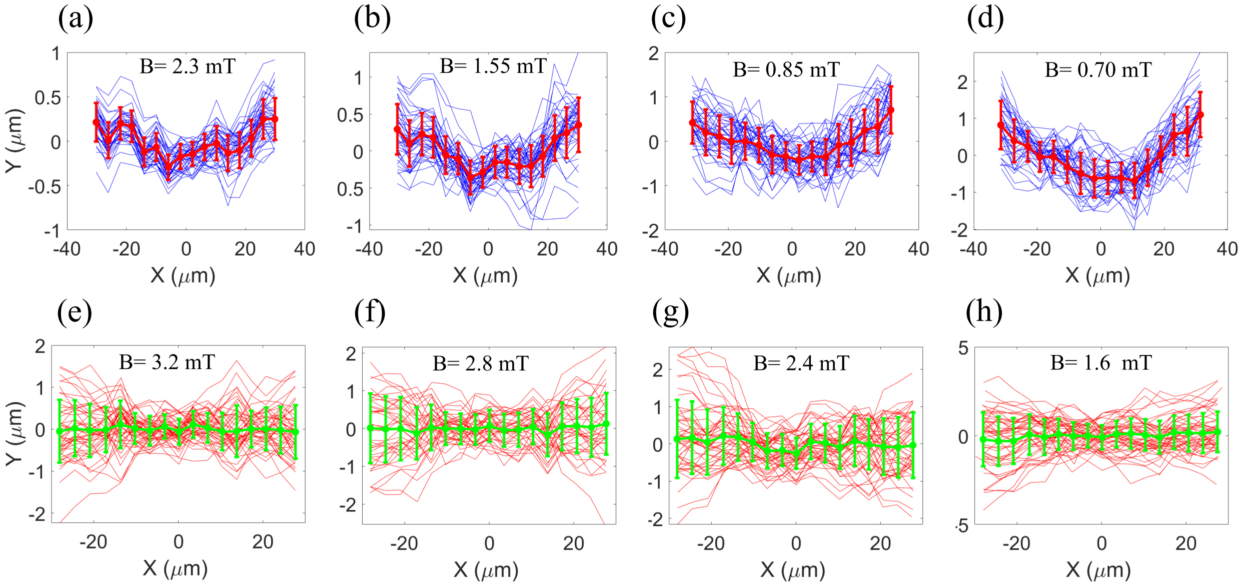

Figure S3: Deflection of the chains presented in Figures 3 and 4 of the manuscript. The deflection curves are shown again here with error bars, which represent the standard deviation of the particle positions. Note also that a smaller y-axis interval is used to better visualize the bending of the chain in the passive case. The y-axis interval is not fixed.

Movie S1: Time series of a self-assembled magnetic chain in a passive bath. A chain of 16 magnetic particles is moving in a motility buffer at a curved liquid-air interface. Magnetic field is $B = 0.7$ mT. The chain exhibits both a center of mass motion and internal fluctuations. In this setup, the magnetic field is applied horizontally, while the tangent gravitational force at the interface points downward, i.e., $\phi = 90°$. The small slope of the interface results in the chain primarily adopting a buckled configuration as it gradually moves downward towards the center of the droplet interface. See Figure 3 (d) in the main text.

Movie S2: Time series of a self-assembled magnetic chain in an active bath. A chain of 17 magnetic particles is immersed in a bacterial bath at a curved liquid-air interface. Magnetic field is $B = 1.6$ mT. The chain exhibits both center of mass motion and internal fluctuations. In this arrangement, the magnetic field is applied horizontally, while the tangent gravitational force at the interface points downward, i.e., $\phi = 90°$. Despite the pronounced fluctuations experienced by the chain in the active bath, it does not maintain a consistent

buckled orientation, which contrasts with the behavior in a passive case. See Figure 4 (d) in the main text.

References

(1) Savino, R.; Fico, S. Transient Marangoni convection in hanging evaporating drops. *Physics of Fluids* **2004**, *16*, 3738–3754.

(2) Lacoste, D.; Brangbour, C.; Bibette, J.; Baudry, J. Thermal expansion within a chain of magnetic colloidal particles. *Physical Review E* **2009**, *80*, 011401.

(3) James, J.; Spittle, J.; Brown, S.; Evans, R. A review of measurement techniques for the thermal expansion coefficient of metals and alloys at elevated temperatures. *Measurement science and technology* **2001**, *12*, R1.
